# Prevalence and Risk Factors of L‐Asparaginase‐Related Thrombosis Among Acute Lymphoblastic Lymphoma Patients in a Resource‐Limited Setup of Sub‐Saharan Region

**DOI:** 10.1002/cnr2.70153

**Published:** 2025-03-06

**Authors:** Amira Abrar Mohammed, Fozia Abdela, Abel Tenaw Tasamma, Yemisrach Kifle Shewangizaw, Mahlet Tsige Weldeamanuel

**Affiliations:** ^1^ Department of Internal Medicine, School of Medicine, College of Health Sciences Addis Ababa University Addis Ababa Ethiopia

**Keywords:** acute lymphoblastic leukemia (ALL), L‐asparaginase, thrombosis

## Abstract

**Background:**

L‐asparaginase is an important component of acute lymphoblastic leukemia (ALL) treatment. However, it is associated with an increased risk of thrombosis which in turn increases the risk of morbidity and mortality and also poses a negative effect on leukemia‐related outcomes.

**Aims:**

To assess the prevalence of thrombotic events and their associated factors in patients with ALL treated with L‐asparaginase‐containing regimens in Tikur Anbessa Specialized Hospital (TASH) from November 2020 to November 2023.

**Methods:**

A single‐center retrospective cross‐sectional study was conducted at TASH. A total of 152 ALL patients who have been treated or were on treatment with L‐asparaginase‐containing regimens from November 2020 to November 2023 were included in the study. Data were collected from the patient's medical records. Data were entered and analyzed with SPSS version 26, and a chi‐square test was used to assess the association of independent variables with the dependent variable. Bivariate and multivariate logistic regression analyses were used to determine the presence of significant correlation between dependent and independent variables and those with *p*‐value of 0.25 were included in the multivariate analysis. For multivariate analysis a *p*‐value < 0.05 was considered as statistically significant.

**Results:**

The median age of the participants was 22.5 years (IQR 18, 30.8), and 59.9% of them were male. The majority (84.2%) of patients were treated with pediatric‐inspired ALL CALGB10403 protocol. Eleven point two percent of the patients developed a documented thrombotic event. All of the events were venous and, cerebral venous thrombosis was the most common site (41.2%). Thrombosis occurred during remission induction in 44.4%. On multivariate logistic regression analysis, age ≥ 40 years and longer time to achieve complete remission (CR) of > 4 weeks (Age ≥ 40 years, *p* = 0.019; adjusted odds ratio [AOR] 10.4, 95% confidence interval [CI] = 1.47–75.0 and, time to achieve CR > 04 weeks *p* = 0.037; AOR 4.8, 95% CI = 1.10–20.72) were significantly associated with increased risk of thrombosis. Patients who developed thrombosis had a statistically non‐significant higher rate of mortality compared with those without thrombosis (47% vs. 41.4%, *p* = 0.618). Around a third (29.4%) of the deaths in the patients with thrombosis were direct effects of the thrombotic event.

**Conclusion:**

This study showed that the risk of L‐asparaginase‐associated thrombosis in resource‐limited settings like ours is comparable with previous reports from other parts of the world, but the mortality directly attributed to thrombosis is remarkably higher. Older age above 40 years and longer time to achieve CR are independent predictors of higher thrombosis risk. Future prospective studies need to look into the contributing factors, and preventive and treatment strategies.

## 1 Introduction

Acute lymphoblastic leukemia (ALL) is the most common pediatric cancer, and it accounts for 20% of adult acute leukemia. The treatment outcome of ALL has improved over the past several years. With current treatment, 98% of pediatric ALL patients achieve complete remission (CR), and long‐term survival reaches ~90% [1]. Despite a comparable CR rate of 90%, the long‐term survival rate remains suboptimal in adults, with only 40%–50% of patients surviving for > 5 years [2].

L‐asparaginase is an important component of most ALL chemotherapy regimens. It has a proteolytic activity that converts asparagine into aspartic acid and ammonia. Asparagine is a non‐essential amino acid that is important for protein synthesis and normal cell growth. A decreased level of asparagine will lead to the activation of the apoptotic pathway. As lymphocytes can't synthesize de novo asparagine, the asparagine depletion associated with asparaginase therapy will lead to apoptosis [3].

Incorporation of L‐asparaginase in ALL treatment protocols has improved complete remission and survival. Several pediatric studies have shown improvements in both CR and survival rates with protocols that incorporate L‐asparaginase [4]. Although the benefit of L‐asparaginase in adults is less studied, recent evidence supports its beneficial effect. This has particularly been demonstrated with the use of pediatric‐inspired protocols in adults, which generally have higher cumulative doses of asparaginase than conventional adult protocols, with reported 5‐year overall survival and event‐free survival rate of 52%–78% [5].

Unfortunately, the use of L‐asparaginase in ALL has been complicated by different peculiar toxicities, one of which is thrombosis. The reported rates of L‐asparaginase‐related thrombosis range from < 5% in the pediatric population to 34% in adults [6]. The main mechanism is said to be inhibition of protein synthesis, leading to decreased levels of anticoagulants, mainly anti‐thrombin. Supporting the role of L‐asparaginase in thrombosis development is the absence of reported thrombosis as a treatment complication in adult protocols that do not contain L‐asparaginase, like hyper‐CVAD [7].

Several risk factors have been associated with increased risk of developing L‐asparaginase‐related thrombosis. Amongst these are older age, T‐cell phenotype, and high‐risk ALL [6, 8]. Other studies have shown an association between the dose and duration of L‐asparaginase therapy and the concomitant drugs administered with L‐asparaginase [9]. On the other hand, measures like FFP supplementation during L‐asparaginase therapy [8, 10] and anti‐thrombin repletion [11, 12] have been shown to decrease the risk of thrombosis.

There is a significant paucity of data regarding L‐asparaginase‐related thrombosis in Ethiopia and Sub‐Saharan Africa settings at large. Thus, we conducted this retrospective study to evaluate the prevalence, risk factors, and outcomes of thrombosis in adults with ALL treated with L‐asparaginase‐containing regimens in our institution.

## 2 Methods

### 2.1 Study Design

An institution‐based retrospective cross‐sectional study was conducted at Tikur Anbessa Specialized Hospital (TASH) from November 2023 till February 2024. All patients with ALL treated at TASH during the study period and who fulfilled the inclusion criteria were included in the study.

### 2.2 Study Area

The study was conducted at TASH, which is the largest tertiary hospital in Ethiopia, found in the capital, Addis Ababa. It has been the only government hospital that provides acute leukemia treatment for many years, until a few other centers have started the service recently.

### 2.3 Study Population

All patients aged ≥ 13 diagnosed with ALL and treated at TASH from November 2020 to November 2023 and who fulfilled the inclusion criteria.

### 2.4 Sample Size and Sampling Procedures

**FIGURE 1:**
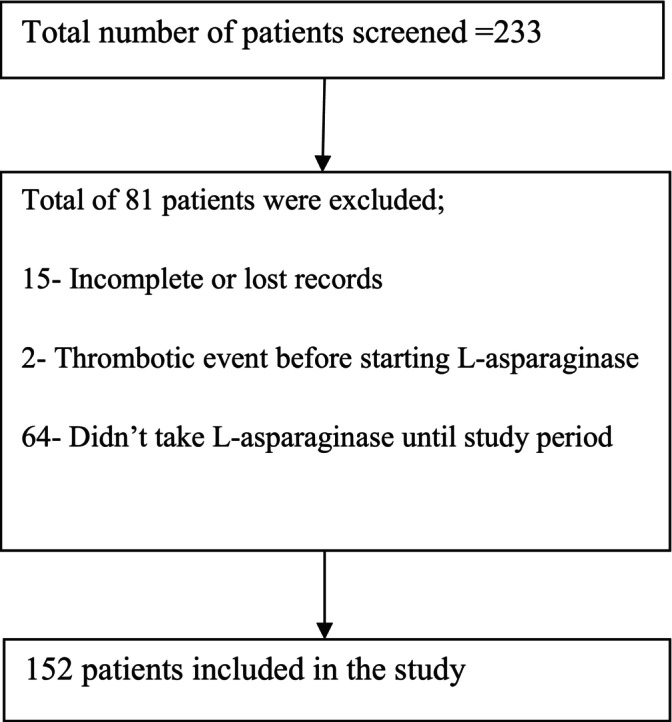
Screened patients for the study.

The sample size was calculated using a single population formula. The population proportion was taken as 10% from a study done in Europe [6]. The calculated sample size is 139; adding 10% non‐responder rate, the final sample size is 153. Among screened patients, 152 fulfilled the inclusion criteria, and all were included in the study. The medical records of patients with a documented diagnosis of ALL were screened from HMIS registries and electronic medical records. 64 patients were excluded because they did not take L‐asparaginase until the study period. Two patients had thrombotic events before starting L‐asparaginase, and 15 patients were excluded for incomplete or lost records. Finally, a total of 152 patients were included in the study. The screening process is described in Figure 1.

### 2.5 Eligibility Criteria

#### 2.5.1 Inclusion Criteria

- Age ≥ 13 years with ALL.
- Morphologic or flow cytometry confirmed diagnosis of ALL.
- Had taken at least one dose of L‐asparaginase therapy.

#### 2.5.2 Exclusion Criteria

- Patients who had confirmed thrombosis before taking L‐asparaginase.
- Patients who had not taken any dose of L‐asparaginase until the study period.
- Patient with lost medical records.

### 2.6 Study Variables

#### 2.6.1 Dependent Variable

- L‐asparaginase‐associated thrombosis

#### 2.6.2 Independent Variables

- Age
- Sex
- Blood group and Rh
- ALL risk group
- Immunophenotype of ALL (if flow cytometry was done)
- Type of L‐asparaginase used
- Total dose of L‐asparaginase taken
- Duration of L‐asparaginase treatment
- Presence of other risk factors for thrombosis (presence of recent surgery, immobilization, indwelling central venous catheter (CVC), current oral contraceptive use, and family history of thrombosis)
- Concomitant treatment with FFP
- Pharmacologic thrombophylaxis

### 2.7 Operational Definitions

• Acute Lymphoblastic Leukemia: Leukemia of lymphocyte progenitor origin that is confirmed by morphology or flow cytometry
• Thrombotic events: Any symptomatic or asymptomatic thrombotic event involving the arterial or venous system and confirmed by appropriate imaging modality.
  ○ For CNS thrombosis confirmation by magnetic resonance imaging (MRI), magnetic resonance venography (MRV), or computed tomography scan
  ○ For pulmonary embolism: Computed tomography pulmonary angiography
  ○ For deep venous thrombosis: Doppler or compressive ultrasound
  ○ For superficial venous thrombosis, ultrasound
  ○ For myocardial infarction confirmed by electrocardiography or cardiac biomarker
• Complete hematologic remission: Leukemic cells not detectable by light microscopy (< 5% blast cells in bone marrow).
• Hematologic relapse: > 5% blast cells in bone marrow/blood.
• High‐risk ALL: Patients with any of the following features:
  ○ High WBC count at diagnosis > 30 000/microL for B‐ALL and > 100 000/microL for T‐ALL
  ○ BCR/ABL‐1 like gene signature
  ○ Progenitor B‐cell immunophenotypes expressed including CD79a, CD19 and cytoplasmic CD22 but not CD10
  ○ > 04 weeks of induction therapy required to attain CR
  ○ Older age > 60 years
  ○ Clonal cytogenetic abnormalities *t*(4;11), *t*(9;22), *t*(1;19)
  ○ MRD: A post‐remission bone marrow MRD level ≥ 10^−3^ using patient‐specific Ig/TCR gene re‐arrangements.
• Intermediate risk: None of the high‐risk criteria are met and age 30–59 years.
• Standard risk: None of the intermediate or high‐risk criteria are met.
• Interruption in treatment of L‐asparaginase: patients who didn't take L‐asparaginase on the protocol schedule date or missed one or more doses.

### 2.8 Data Collection Procedure and Tools

A structured questionnaire was used to collect the data, and data collection was conducted by the primary investigator. Data was collected from patients’ medical records, both paper and electronic, from December 2023 until February 2024.

### 2.9 Data Quality Assurance

The data was assessed for accuracy and completeness by the primary investigator before proceeding to data analysis. A pre‐test was done using 5% of the sample size to clarify any queries and inconsistencies on the checklist before official data collection was started.

### 2.10 Data Analysis

After quality was assessed for accuracy, data was entered into the SPSS version 26 program. It was then recorded and analyzed. Descriptive statistics were computed for each variable. Depending on the normality of the data, the mean, median, interquartile ratio, and standard deviation were computed and summarized in tables and graphs.

A chi‐square test was done to assess the relationship between dependent and independent variables. A bivariate test was done to calculate the crude odds ratio. Those variables with a *p*‐value of < 0.25 based on the Hosmer and Lemshow test were included in a multivariate analysis. AOR was analyzed, and a *p*‐value of < 0.05 was taken as statistically significant for the multivariate analysis. A 95% confidence interval was also calculated for the variables.

### 2.11 Ethical Consideration

Ethical clearance was obtained from the Research and Ethics Review Committee of the Department of Internal Medicine, and then from the Addis Ababa University, College of Health Sciences Ethical Review Board. The collected data was kept confidential and was used for study purposes only.

## 3 Results

### 3.1 Socio‐Demographic Characteristics

A total of 152 patients were included in this study. The median age of the participants was 22.5 years (IQR 18, 30.8) and close to half of them (47.4%) were in the age group of 18–29 years. Males predominated, accounting for 59.9% of the patients. Table 1 summarizes the socio‐demographic and baseline characteristics of the study participants.

**TABLE 1:** The socio‐demographic and baseline characteristics of the study participants.

Variable		Frequency	Percent (%)
Age in years	< 18	36	23.7
	18–29	72	47.4
	30–40	21	13.8
	> 40	23	15.1
Sex	Male	91	59.9
	Female	61	40.1
Blood group and RH	A positive	47	30.9
	B positive	38	25
	AB positive	4	2.6
	O positive	50	32.9
	A negative	3	2.0
	AB negative	1	0.7
	O negative	9	5.9
Blood group category	O blood group	59	38.8
	Non O blood group	93	61.2

### 3.2 Disease Characteristics of the Study Participants

Flow cytometry for leukemia diagnosis was done only for 32 (21%) of the participants; accordingly, 16 patients each were B‐ALL and T‐ALL, respectively. Philadelphia chromosome status was checked for 109 (71.7%) patients, and 17 of them (15.5%) tested positive.

The median baseline WBC count was 27 200/μL. Almost half of the study participants had an initial WBC of less than 30 000. The presentation WBC count was more than 100 000 in 28.3% of the participants. When risk stratification was made based on WBC count at presentation according to the criteria mentioned in the operational definition, 25% of the B‐ALL & 56.2% of the T‐ALL patients fell into high‐risk group.

The commonest blood group was O positive, accounting for 32.9%, followed by A positive (30.9%). Overall, 38.8% of participants had a group O blood type, while 61.2% had a non‐O blood type. Details of the disease characteristics of the study participants are summarized in Table 2.

**TABLE 2:** Acute lymphoblastic leukemia characteristics of the study participants.

Variable		Frequency	Percent (%)
ALL subtype	B‐ALL	16	10.5
	T‐ALL	16	10.5
	Not known	120	79
Ph chromosome	Positive	17	11.2
	Negative	92	60.5
	Not known	43	28.3
WBC at presentation (cells/μl)	< 30 000	77	50.7
	30 000–100 000	32	21.1
	> 100 000	43	28.3
Median presentation WBC (IQR)	27 200 cells/μl (4000,109 000)		

### 3.3 Treatment Characteristics of the Study Participants

The majority of the patients (84.5%) were treated with the pediatric‐inspired CALGB 10403 protocol and the rest with the adult GALGB 8811, all of which contain L‐asparaginase as a treatment component. Overall, 91 (59.9%) patients achieved CR within 4 weeks of remission induction, while 11 (7.2%) required extended remission induction for two more weeks. In 50 (32.9%) patients, CR was not assessed because the patients died or went against medical advice during remission induction or thereafter before the assessment of CR was done.

44% (*n* = 67) of the study participants had interruptions in L‐asparaginase treatment, hepatotoxicity being the commonest (40.3%) reason.

Only 5 patients (3.3%) received pharmacologic thromboprophylaxis at one point during the treatment period. Four (2.6%) patients received FFP transfusion. Table 3 summarizes the treatment characteristics of the participants.

**TABLE 3:** Risk factor for thrombosis and ALL‐related treatment.

Variable		Frequency	Percent (%)
Documented baseline coagulation	Yes	35	33
	No	117	77
PT	Median (IQR)	13.4 (11.9,15.1)	
INR	Mean ± SD	1.21 ± 0.17	
PTT	Median (IQR)	31 (27.9,35.8)	
Patients' phase of treatment at the time of data collection	Remission induction	63	41.4
	Consolidation (EI)	28	18.4
	Interim maintenance/CNS prophylaxis	10	6.6
	Delayed intensification	9	5.9
	Long‐term maintenance	42	27.6
Time to achieve CR (wks)	≤ 04	91	59.9
	> 04	11	7.2
	CR not assessed	50	32.9
Total dose of L‐asparaginase (in 1000 IU/m2) median (IQR)		39 (24108)	
Was L‐asparaginase interrupted?	Yes	67	44.1
	No	85	55.9
Reason for interruption (*n* = 67)	Hepatotoxicity	28	41.7
	Thrombotic event	10	14.9
	Medication unavailability	17	25.3
	Others	14	20.8
Did patient receive pharmacologic thrombophylaxis?	Yes	5	3.3
	No	147	96.7
Did patient receive FFP transfusion?	Yes	4	2.6
	No	148	97.4
ALL treatment regimen	CALGB 8811	24	15.8
	CL10403 regimen	128	84.2

### 3.4 L‐Asparaginase Associated Thrombotic Events

None of the participants had a thrombosis history in the past. Five patients had documented traditional risk factors for thrombosis. These were a recent cesarean section in one patient and OCP use for abnormal uterine bleeding and immobilization due to ICU admission in two patients each. However, none of these patients developed thrombosis.

**FIGURE 2:**
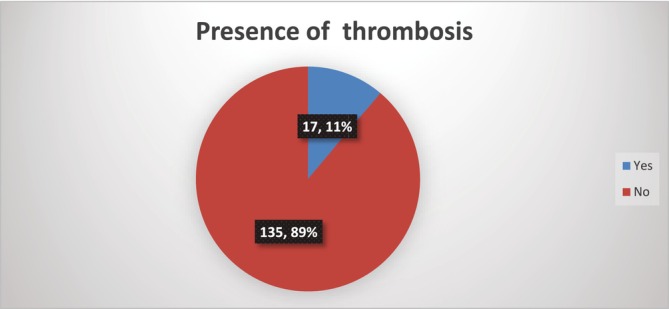
The prevalence of thrombosis among ALL patients.

Among the 152 ALL patients treated with L‐asparaginase‐containing chemotherapy regimens in this study, 17 (11.2%) developed a thrombotic event (see Figure 2). All of the thrombotic events were venous, and all of them were symptomatic, except one patient who had asymptomatic internal jugular vein (IJV) thrombosis.

44.4% of the thrombotic events occurred during remission induction, followed by 33.3% during early intensification (EI).

Cerebral vein thrombosis (CVT) was the most common (41.2%, *n* = 7) site of thrombosis. It was confirmed by MRV in 4 patients, while the diagnosis was made by MRI and contrast CT in two and one patient, respectively. Lower limb DVT was seen in 6 patients (35.3%), one of whom also had a concomitant CTPA‐confirmed pulmonary embolism (PE). Two patients had an upper limb DVT. One patient had IJV thrombosis, and another one had a superficial vein (cephalic) thrombosis.

Among CVT patients, seizure was the commonest manifestation, followed by decreased mentation. One patient presented with a headache and cranial nerve palsy (ptosis). All of the extremity DVTs presented with limb swelling and pain. The one PE patient presented with sudden onset respiratory distress. All patients had a determination of coagulation at the time of thrombosis before the initiation of anticoagulation. Of these patients, 88.2% had a normal coagulation profile. Details of the thrombotic events are summarized in Table 4.

**TABLE 4:** Characteristics of thrombotic event.

		Frequency	Percent (%)
Type of thrombosis	Venous	17	100
	Arterial	0	0
Site of venous thrombosis	CVT	7	41.2
	Lower limb DVT	6	35.3
	Upper limb DVT	2	11.8
	PTE	1	5.9
	Others	2	11.8
VTE Symptoms	Seizure	5	
	Decreased mentation	3	
	Extremity pain	7	
	Swelling	8	
	Cranial nerve palsy	1	
	Respiratory distress	1	
	Asymptomatic	1	
Median (IQR) time from initiation of L‐asp to thrombosis in days		37 (24,60.5)	
Doses of L‐asp taken before onset of thrombosis(1000 U/m2) median (IQR)		36 (33,63)	
Phase of treatment at which the thrombosis occurred	Remission induction	8	44.4
	Consolidation 1 (EI)	6	33.3
	Consolidation 2 (Interim maintenance)	1 (5.6)	5.6
	Delayed intensification (DI)	2	11.1
	Long‐term maintenance	1	5.6
Coagulation profile at time of thrombosis	PT median (IQR)	14 (12.4,14.9)	
	PTT	30.3 ± 5.6	
	INR	1.21 ± 0.17	

### 3.5 Treatment Outcome of Characteristics of the Thrombosis

All patients with thrombosis received anticoagulation. Unfractionated heparin was used in 58.8% of cases. Two patients received warfarin. Rivaroxaban and enoxaparin were used in 29.4% and 11.8% of the patients, respectively. There was an overlap between different anticoagulants, and patients were switched from one anticoagulant to another.

Among the 17 patients with thrombosis, nine (52.9%) developed thrombosis‐related complications. The complications were permanent physical impairment (ptosis) from CVT in one patient, ICU admission in four patients (all of whom subsequently died), and death without ICU admission in another four patients. The cause of death in the four ICU‐admitted patients was increased ICP in two CVT patients and respiratory failure in the other two patients. One of these patients with respiratory failure had CTPA‐confirmed PE, and another one had clinically suspected PE with a confirmed lower limb DVT. The cause of death in the remaining four patients was intracranial hemorrhage in one patient with CVT due to severe thrombocytopenia, sudden cardiac arrest due to massive PE in a patient with CVT, and sepsis in the other two. The treatment of L‐asparaginase‐associated thrombosis is detailed in Table 5.

**TABLE 5:** Treatment of L‐asparaginase‐associated thrombosis.

Variable		Frequency	Percent (%)
Mechanism of management			
Was anticoagulation administered	Yes	17	100
	No	0	0
Types of anticoagulation (*n* = 17)	Warfarin	2	11.8
	UFH	10	58.8
	Enoxaparin	2	11.8
	Rivaroxaban	8	29.4
Duration of anticoagulation	Median (IQR)	60 (4.5120)	
Thrombosis‐related complication (*n* = 17)	Yes	9	52.9
	No	8	47.1
Types of complications (*n* = 10)	Death	4	44.4
	Required ICU admission	4	44.4
	Permanent physical impairment	1	11.1
L‐asparaginase resumed after thrombosis (*n* = 17)	Yes	3	17.6
	No	14	82.4

### 3.6 ALL‐Related Outcomes of the Patients With Thrombosis

**FIGURE 3:**
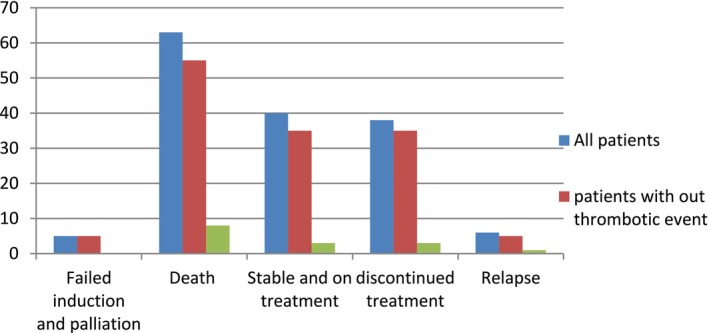
The ALL‐related outcomes of the study participants.

Among the 17 patients with thrombosis, 8 (47%) died, while five patients (29.4%) discontinued treatment (see Table 6). One patient had a relapse and was referred abroad for further treatment. Three (17.6%) are stable and currently on follow‐up taking long‐term maintenance therapy. There was not a statistically significant difference in ALL‐related outcomes in the patients who did and did not develop thrombosis. Figure 3 compares treatment outcomes between patients who developed thrombosis and those without thrombosis.

**TABLE 6:** ALL‐related outcomes of patients.

ALL‐related outcomes of the patients	Total		Thrombotic event				*p*
			Yes		No		
	*n*	%	*n*	%	*n*	%	
Failed induction and palliation	5	3.3	0	0	5	5.2	0.42
Death during RI or afterwards	63	41.4	8	47	55	40.7	0.618
Still on treatment and stable	40	26.3	3	17.6	37	25.9	
Discontinued treatment	38	25	5	29.4	33	24.4	
Relapse	6	3.9	1	5.9	5	3.7	0.664

### 3.7 The Chi‐Square Relation Between Thrombotic Events and Independent Variables

The chi‐square test revealed that age (*p =* 0.131), blood group (*p =* 0.057), and duration to achieve CR (*p* = 0.004) were associated with the development of thrombotic event in the chi‐square test, as shown in the Table 7 Furthermore, while B‐ALL was associated with higher thrombotic risk, use of pharmacologic prophylaxis was associated with a lower risk of thrombosis.

**TABLE 7:** The chi‐square relation between the thrombotic events and independent variables.

Variable		Thrombosis event		Chi‐square value	*p*
		Yes	No		
Age in years	< 18	2	34	5.62	0.131
	18–29	6	66		
	30–40	4	24		
	> 40	5	11		
Sex	Male	8	83	1.307	0.253
	Female	9	52		
ALL sub‐type	B‐ALL	4	12	2.13	0.14
	T‐ALL	1	15		
PH chromosome	Positive	3	14	0.906	0.341
	Negative	9	83		
Blood group	O	3	56	3.612	0.057
	Non‐O	14	79		
Duration to achieve CR	4 weeks	11	80	8.26	0.004
	> 4 weeks	5	6		
Regimen	CALGB	5	19	2.671	0.102
	Pediatric‐inspired regimen	12	115		
Pharmacologic prophylaxis	Yes	3	132	4.322	0.038
	No	2	15		
Interruption in L‐asparaginase	Yes	80	6	3.530	0.060
	No	55	11		

On the other hand, Philadelphia chromosome status (*p =* 0.341), sex (*p =* 0.253), use of FFP (*p =* 0.472), presentation WBC count (*p =* 0.935), phase of treatment (*p* = 0.742), and total dose of L‐asparaginase (*p =* 0.298) all had no significant correlation with thrombosis development.

### 3.8 Determinant Factors for Thrombotic Events

A bivariate regression analysis was done for the independent variables, and if a statistically significant association was seen based on the Hosmer‐Lemeshow test, they were included in the multivariate regression analysis. We didn't include the type of regimen on multivariate analysis, despite it having an association in the bivariate logistic regression, as it overlaps with age. All patients' ≥ 40 years were treated with the adult CALGB 8811 regimen. Similarly, we didn't include ALL subtypes on the multivariate regression analysis, despite it having a significant association in the bivariate regression analysis, as only 21% of the study participants had flow cytometry done.

As described in Table 8, study participants' age, blood group, time to achieve CR, and use of prophylactic anticoagulation were significantly associated with thrombotic events in the bivariate logistic regression.

**TABLE 8:** The bivariate and multivariate regression analysis for the association between independent variables and thrombotic events among ALL patients.

Variable		Thrombosis event		*p*	COR (95% CI)	*p*	AOR (95% CI)
		Yes	No				
Age in years	< 18	2	34	1		1	
	18–29	5	66	0.606	1.5 (0.30, 8.07)	0.671	1.5 (0.25, 8.48)
	30–40	4	24	0.130	4.0 (0.66, 24.06)	0.501	2.1 (0.24, 19.1)
	> 40	6	11	0.080	4.7 (0.83, 26.81)	0.019	10.4 (1.47, 75.0)
Blood group	O	3	56	1		1	
	Non‐O	14	79	0.070	3.31 (0.91, 12.05)	0.126	3.2 (0.72, 14.01)
Time to achieve CR	4 weeks	11	80	1		1	
	> 4 weeks	5	6	0.009	6.06 (1.58,23.23)	0.037	4.8 (1.10, 20.72)
Pharmacothrombo phylaxis	Yes	2	3	1		1	
	No	15	132	0.063	0.17 (0.03,1.10)	0.176	0.21 (0.02,2.04)

In the multivariate regression analysis, patients aged ≥ 40 years had a 10.4 times increased risk of thrombotic event compared with patients aged < 18 years (AOR = 10.4, 95% CI = 1.47, 75.0). Similarly, patients who required > 4 weeks of remission induction therapy to achieve CR had a 4.8‐fold higher thrombotic events when compared with those who achieved CR within 4 weeks (AOR = 4.8, 95% CI = 1.10, 20.72).

## 4 Discussion

L‐asparaginase is an important component of chemotherapy regimens used for ALL treatment. However, it is associated with a significant risk of thrombosis. In this study, we analyzed the prevalence of thrombotic events and their determinant factors in ALL patients with an age greater than or equal to 13 years treated with L‐asparaginase‐containing regimens from November 2020 to November, 2023 in TASH, a tertiary care hospital in Addis Ababa, Ethiopia.

The prevalence of L‐ asparaginase‐associated thrombosis in this study is 11.2%. This is comparable with a multicenter study done in Europe that reported a prevalence of 10% [13]. It is also similar to a report from a university‐based cancer center in the United States of America (USA), which was 11.2% [14]. The later study included adult ALL patients treated with PEG‐asparaginase, unlike our study where all of the patients were treated with *Escherichia coli* L‐asparaginase. On the other hand, the prevalence of thrombosis in our study is lower than the Dana‐Faber Consortium study, which reported 34% and 5% prevalence in adult and pediatric patients respectively [6]. This could be due to the differences in the characteristics of patients, as they defined adults as aged > 18 years whereas we included patients aged ≥ 13 years, according to our hospital's admission policy. Otherwise, 23.7% of our patients were < 18 years old and would be included in the pediatric group according to the Dana‐Faber Consortium study. In addition, the fact that none of our patients had an indwelling catheter, a known risk factor for thrombosis, could have contributed to the lower occurrence of thrombosis in our setting. The prevalence was also much lower than the report from Ohio University of 41% among adolescents and young adults (AYA) treated with pegylated asparaginase as part of a pediatric‐inspired regimen [14]. This study reported a particularly high thrombosis prevalence, which was significantly high even when compared with other reports of thrombosis in AYAs treated with pediatric‐inspired protocols (e.g., 5% in the C10403 protocol). The authors mentioned that the reason for this was unclear and cannot be explained by the presence of an indwelling CVC, as only 11% of the events in the study were catheter‐related compared with 50% in the C10403 study. Variation in the reported thrombosis prevalence among studies could be due to many factors, such as differences in treatment protocol, genetic variation, use of anti‐thrombin infusions, and the presence of other risk factors for thrombosis.

CVT was the most common site of thrombosis in our study, which is in line with the findings of other studies [15, 16, 17]. However, there are also studies that have found upper extremity DVT, which was low (11%) in our study, to be the commonest site of thrombosis [6, 18]. The absence of CVC use in our setup might contribute to the lower upper extremity DVT.

Thrombotic events occurred most frequently during remission induction therapy in our study (44.4%), though the association was not statistically significant (*P* = 0.74). This has also been reported by other studies. A meta‐analysis of 17 prospective pediatric studies on the thrombotic complications of ALL found 61 events in 1280 pts. during induction, corresponding to a thrombosis incidence rate (IR) of 4.8% versus 12 events in 609 patients, corresponding to an IR of 2% in later phases of treatment [17]. Similarly, a majority (72%) of the patients experienced the initial venous thromboembolic event (VTE) during the induction phase of ALL treatment in the report by Brynne Underwood et al. [14].

The time from the administration of the first dose of L‐asparaginase to the development of thrombosis was 37 days in our study, which was shorter than the Dana‐Farber report of 3.5 months [6] but longer than the Dutch–Belgian HOVON‐37 study of 23 days [10].

Age is an established risk factor that has consistently been associated with increased risk of L‐asparaginase‐related thrombosis. We found that there is a statistically significant increased risk of thrombosis in patients ≥ 40 years of age with an AOR of 10.4 (95% CI = 1.47, 75.0). A Korean study found a 2–3 times increased risk of thrombosis in adults > 18 years of age [19]. The Dana‐Faber Consortium study identified the frequency of VTE to increase along the age continuum from 2% (ages 0–5 years) to 20% (ages 11–14 years) to 42% (age > 30 years) [6].

We found a longer time to achieve CR of > 04 weeks to be significantly associated with an increased risk of thrombosis with an AOR of 4.8 (1.10, 20.72). Though we have not seen similar reports from others, there are studies that found high‐risk ALL to have an increased risk of thrombosis development [6, 8]. In a Canadian pediatric study, six out of seven patients who developed thrombosis (86%) had a high‐risk ALL [8]. We speculate that as longer time to achieve CR of > 04 weeks is one of the criteria for high‐risk ALL, it could be the reason for the noted increased thrombosis risk in this group in our study.

In our study, the dose of L‐asparaginase didn't show an association in the chi‐square test and was not included in the multivariate analysis. This could be due to interruption of L‐asparaginase in patients who developed thrombosis when compared to longer duration and cumulative dose in those who did not develop such complication. On the other hand, non‐O blood type was associated with higher thrombosis risk with a COR of 3.31 (0.91, 12.05) on bivariate analysis, though it lost its significance on multivariate analysis (*p* = 0.13). Previous studies also showed an association with non‐O blood group and higher L‐asparaginase‐related thrombosis risk, a potential explanation being higher von Willebrand factor and factor VIII levels associated with non‐O blood types [20, 21].

Though the higher prevalence of thrombosis in B cell ALL patients in our study contradicts with the previous reports from others [6, 20], it is difficult to make any conclusion from our finding as only a few patients had a flow cytometry done. While prior studies have suggested a link between BCR‐ABL negativity and increased thrombosis risk (potentially mitigated by TKI therapy), this study did not observe a statistically significant association.

Several preventive measures to decrease the risk of L‐asparaginase‐related thrombosis have been tried, such as administration of FFP, anti‐thrombin repletion, and use of prophylactic low molecular weight heparin (LMWH). A Dutch study has shown that the administration of FFP on the days of scheduled L‐asparaginase therapy can significantly lower VTE incidence: 6% versus 19%; AOR 0.28 [20]. Our study also found a statistically non‐significant (*p =* 0.472) lower thrombosis risk in the patients who received FFP, but it should be noted that the number of patients who received FFP was only four. Similarly, the use of pharmacologic thrombophylaxis has been shown to decrease thrombosis risk, especially in the pediatric population [22]. We also found a lower thrombosis risk in the patients who received unfractionated heparin prophylaxis with a COR of 0.17 (0.03, 1.10), but it wasn't statistically significant on multivariate analysis, which could be explained by the very low number of patients who got the prophylaxis (only 5 out of 152).

Similar to the Dana‐Farber Consortium report, we found no statistically significant mortality difference between the patients with and without thrombosis (47% vs. 41.4%, *p* = 0.618). However, it is striking that there was a significant percentage of death (29.4%) directly related to thrombosis in our study. There were no deaths directly related to thrombosis in the Dana‐Farber report [6]. This is most likely due to the suboptimal care of critically ill patients in our setup, especially when admitted in ICU, due to resource limitations.

This study, to our knowledge, is the first of its type that assessed the thrombotic complications associated with L‐asparaginase therapy in ALL patients treated in resource‐limited setups of Sub‐Saharan Africa. Thrombosis in Sub‐Saharan Africa is a complex issue with many factors contributing to its prevalence and clinical features. Some risk factors, like infections, including a very high prevalence of HIV and nutritional deficiencies, are important factors that need special emphasis in this part of the world. Though data on L‐asparaginase‐related thrombosis in the Sub‐Saharan Africa region is almost absent, there are studies that showed a very short survival of patients with cancer‐related thrombosis. Among factors contributing to high mortality could be suboptimal anticoagulation due to lack of coagulation profile monitoring, limitations in accessing blood products like platelet transfusion, and poor access to ICU care. We believe our study gives an insight into the burden of L‐asparaginase‐related thrombosis and the factors contributing to it in this poorly represented part of the world. However, our study has its own limitations. First, since it is retrospective, it is difficult to assess detailed risk factors and laboratory parameters, including coagulation profiles. Second, due to missed medical records, not all patients treated during the study period were included in the study. Third, for some variables, like the use of FFP and prophylactic anticoagulation, the number of patients who received these treatments was very small, which might affect the correlation. Due to financial constraints, flow cytometry and cytogenetic studies were not assessed in a significant proportion of the patients, which could affect the study outcome. Coagulation profiles were not assessed closely in our patients because of availability and cost issues. Because of the unavailability of CVCs in the government setup and the high cost and inconsistent accessibility in the market, patients with ALL in our setup take chemotherapy medications via peripheral venous lines. This might be the reason for the lower incidence of catheter‐related thrombosis and upper extremity DVT in our study. Finally, the study is an institution‐based, single‐centered study, the result of which may not be representative of the general population.

## 5 Conclusion

We identified that there is a significant risk of thrombosis related to L‐asparaginase therapy in ALL patients in a resource‐limited setup of ours. Older age above 40 years and longer time to achieve CR of > 04 weeks were independent factors associated with increased risk of thrombosis. An important finding of this study is that the development of thrombosis will significantly contribute to mortality, as evidenced by the death of nearly a third of the patients who developed thrombosis because of a direct thrombosis effect. We recommend future large‐scale prospective studies to look deep into the contributing factors for L‐asparaginase‐related thrombosis and to optimize treatment and prevention strategies in resource‐limited settings.

## Author Contributions

Conceptualization: A.A., A.T.T., and F.A. Methodology: A.A., A.T.T., and Y.K.S. Investigation: A.A., M.T.W., and Y.K.S. Formal analysis: A.A., M.T.W. and A.T.T. Writing – original draft: A.A. Writing – review and editing: F.A. and A.T.T. Supervision: F.A.

## Ethics Statement

Ethical approval was obtained from the Institutional Review Board (IRB) of Addis Ababa University (AAU), Department of Internal Medicine. Written or verbal consent was not required as the study only used secondary data.

## Conflicts of Interest

The authors declare no conflicts of interest.

## Data Availability

The data that support the findings of this study are available from the corresponding author upon reasonable request.
